# Addressing the Barriers to Clinical Trials Accrual in Community Cancer Centres Using a National Clinical Trials Navigator:A Cross-Sectional Analysis

**DOI:** 10.1177/10732748221130164

**Published:** 2022-09-27

**Authors:** Caroline Hamm, Dora Cavallo-Medved, Devinder Moudgil, Lee McGrath, John Huang, Yueyang Li, Tyler W. Stratton, Tyler Robinson, Krista Naccarato, Stephen Sundquist, Janet Dancey

**Affiliations:** 1 8637 University of Windsor, Windsor, ON, Canada; 2 Western University, Windsor, ON, Canada; 3 Windsor Cancer Research Group, Windsor, ON, Canada; 4 WE-SPARK Health Institute, Windsor, ON, Canada; 5 Invest WindsorEssex, Windsor, ON, Canada; 6 194075 Windsor Regional Hospital, Windsor, ON, Canada; 7 Canadian Cancer Clinical Trials Network, Windsor, ON, Canada; 8 Queen’s University, ON, Canada

**Keywords:** clinical trials, accrual, challenges, barriers, navigator

## Abstract

**Introduction:**

Clinical trials, although academically accepted as the most effective treatment available for cancer patients, poor accrual to clinical trials remains a significant problem. A clinical trials navigator (CTN) program was piloted where patients and/or their healthcare professionals could request a search and provide a list of potential cancer clinical trials in which a patient may be eligible based on their current status and disease.

**Objectives:**

This study examined the outcomes of a pilot program to try to improve clinical trials accrual with a focus on patients at medium to small sized cancer programs. Outcomes examined included patient disposition (referral to and accrual to interventional trials), patient survival, sites of referral to the CTN program.

**Methods:**

One 0.5 FTE navigator was retained. Stakeholders referred to the CTN through the Canadian Cancer Clinical Trials Network. Demographic and outcomes data were recorded.

**Results:**

Between March 2019 and February 2020, 118 patients from across Canada used the program. Seven per cent of patients referred were enrolled onto treatment clinical trials. No available trial excluded 39% patients, and 28% had a decline in their health and died before they could be referred or enrolled onto a clinical trial. The median time from referral to death was 109 days in those that passed.

**Conclusion:**

This novel navigator pilot has the potential to increase patient accrual to clinical trials. The CTN program services the gap in the clinical trials system, helping patients in medium and small sized cancer centres identify potential clinical trials at larger centres.

## Introduction

Currently, clinical trials remain the gold standard to establishing new and reliable treatment options yet it is estimated that only 3% of adults with cancer participate in clinical trials. Further, members of racial and ethnic minorities, elderly and low-income individuals, and people who live in rural areas remain historically underrepresented in trial populations. (EDICT, 2008) The majority of cancer patients are treated in community settings, whereas the majority of cancer patients who enroll in clinical trials are treated within academic settings.^1^

Centres that participate in trials report to have improved patient outcomes.^2^ Participation on clinical trials provide early access to promising and potentially better treatments, and some data suggest that standardized patient management may improve survival.^3-7^ It has been demonstrated that Adolescent and Young Adult (AYA) populations who have shown the lowest accrual to trials, also have the lowest rate of improvement in overall survival over time.^8^

**Figure 1.**
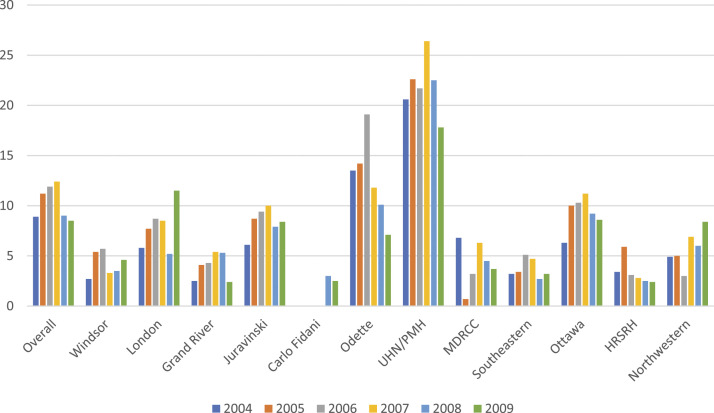
Percent of total number of cancer patients entering clinical trials by individual cancer centres in Ontario, Canada between 2004 to 2009 inclusive(2). These numbers have not changed appreciably in the following years with 7.2% overall patients accrued between 2010 and 2017.

In Ontario, Canada, 24 centres offer publicly funded, specialized cancer care to its 15 million residents. The number of clinical trials offered by these centres ranges from 1 to 350 per site. A cancer patient in Ontario is eligible to enter any trial offered within the province without cost. The average clinical trials accrual for cancer patients across Ontario centres is 7%, with smaller centres averaging 3-5%, and larger centres averaging 20% of their patients enrolled on a trial. At Princess Margaret Hospital (PMH), the largest cancer research hospital in Ontario, 21.9% of patients enter clinical trials. (Figure 1)^9^ In contrast, MD Anderson Cancer centre, one of the largest cancer centres in the USA placed 5% of their patients on a clinical trial.^10^ This may demonstrate a difference between the American and Canadian health care system. Patients in Ontario, Canada are free to travel between cancer centres in Ontario without concern regarding financial coverage of care. In the United States, the patient journey is much more complex, with variable coverage of care in clinical trials.^11^ This factor would have significantly less effect in Canada because of the publicly funded health care. This may explain why the accrual to clinical trials is so much higher in the larger centres in Canada that have more clinical trial opportunities for patients. Statistics that indicate that relatively fewer patients at smaller centres enter clinical trials don’t account for those who are referred to larger centres. Since the proportion of patients entering trials at the larger centres who may be coming from the smaller centres is not collected.

Generally, the causes of poor accrual include low physician engagement, low patient interest, lack of trial availability, unnecessarily strict eligibility criteria and poor site selection. Lack of trial availability is the most frequent barrier to patients interested in entering trials, especially those patients who are treated in smaller centres.^12^ Smaller centres tend to run fewer clinical trials for various reasons including site selection by sponsor, logistical support required for trial complexity, therapeutic or diagnostic technologies necessary for patient care, and scientific or ethical scrutiny by the local research committee and institutional review board.^13^ Even though patients in Canada have access to a wider range of clinical trials by open access to the higher numbers of trials being run at larger centres, referral from smaller centres to larger centres for clinical trials is limited by a lack of infrastructure or capacity to support this process. Patients lack the expertise to search for the appropriate trial, physicians lack the time to search for a trial outside of their centre, and there are no other dedicated personnel for this endeavor.

### Strategies to Improve Accrual to Cancer Clinical Trials

Suggested strategies for improving clinical trials accrual include health care professional (HCP) remuneration, communication and education, enhancing patient education, improving the critical assessment of trial workload before opening, and implementing clinical trials navigators (CTN).^14,15^ Of these, remuneration for patients, multi-disciplinary case rounds discussion^16^ and implementation of clinical trialist performance standards that hold investigators to accrual standards have led to improved accrual.^14^ Patient-centric interactive educational tools^17^ and web-based strategies, such as Craigslist have also been used successfully.^18^ Finally, CTN strategies including pharmaceutical company-based navigators, cancer-centre-based navigators and national approaches have been explored. Between 2007 and 2010, the American Cancer Society (ACS) and Coalition of Cancer Cooperative Groups (CCCG), provided a national clinical trial service focused on providing information, matching and eligibility support.^19^ The service included a website that patients could visit and a call center whereby patients could speak directly with a clinical trial expert. Eleven per cent of patients using this service enrolled on a clinical trial.^19^ With the exception of the ACS navigator program, none of the strategies focused on resolving the problem of patients navigating between the smaller community hospitals and the larger academic hospitals. Because of above mentioned public health care system in Canada, and its implications for clinical trials accrual, we suggest that a Clinical Trials Navigator (CTN) has the potential to be successful in Canada. In 2019, national the Clinical Trial Navigator pilot program, sponsored by the Canadian Cancer Clinical Trials Network (3CTN), was established to address this issue by supporting Canadian patients to find eligible clinical trials outside of their resident centres.

There are a number of current strategies that are working to address the under-represented population of patients in smaller cancer centres. The NCI’s National Clinical Trials Network, the NCI Community Oncology Research Program, Sarah Cannon Research Institute and US Oncology share some infrastructure that allow trials to be opened more quickly. The American Cancer Society Cancer Action Network and the MITRE Corporation are developing and piloting a tool to integrate electronic medical records to help identify patients with trial eligibility.^20^

## Methods

Here, we perform a cross-sectional analysis of 118 consecutive patients who used this Canadian Clinical Trials Navigator is studied in this paper between March 3, 2019 and March 12, 2020. Patients, their families or their health care professionals could refer to this program through the Canadian Cancer Clinical Trials Network (3CTN). The only awareness campaign was held at 2 national 3CTN meetings, and word of mouth. The method of referral was either through the 3CTN website (https://3ctn.ca/for-patients/clinical-trials-nav/) or directly by email (clinicaltrialsnavigator@wrh.on.ca).

Case data recorded included patient demographics and pertinent eligibility criteria, including type and stage of cancer, number and types a of previous lines of therapy and outcomes. Data collected includes: date of referral to the CTN program, date documents were received, number of potential clinical trials, number of eligible trials, patient disease site and stage (AJCC 7 stage, or relapsed/refractory), date first report sent to patient, patient’s home cancer centre, site that patient was referred to for the clinical trial, and if available, patient enrollment status on the clinical trial and follow-up comments on the individual patient. This information was collected from the patient’s referral form and/or from the patient medical chart, obtained from the home medical department after patient consent. All patient information was de-identified to ensure that the identity of any person could not be ascertained in any way. The only patient identifier was a study specific identifier ie CTN 1, CTN 2, etc. All patients that used the CTN program were included consecutively. The referring patient’s cancer centre and trial referral site were tracked, along with whether the referral was generated by the patient’s physician or the patient self-referred. Patient feedback on the program was recorded using informal interview and unsolicited comments. Physician engagement is measured by informal interview, frequency of referrals to the CTN, and frequency of patient referral to a clinical trial. In 2019, 3CTN launched the Clinical Trials Navigator program. This program focuses on helping those patients and health care professionals who may require support in finding suitable clinical trial options outside of their own centres. One 0.5 FTE CTN was hired to support the program. Knowledge of the service is promoted on the 3CTN website and presented to cancer centre trial units, patient partners and other stakeholders at 3CTN national meetings. Patients, their designated family member or their health care professional could access the program through the 3CTN website. https://3ctn.ca No other marketing for this program was carried out because of the limited human resources at that time.

Following a documented consent process, dedicated and trained staff receive referrals, obtain and review patient medical records, determine eligibility criteria, and search clinical trial registries. A tailored report is then provided to patients and/or their physicians summarizing trial options across Canada. As this pilot program was situated in a medium sized cancer centre, and received minimal advertisement, the host site was expected to be a heavier user of the program, with a concern of single site bias in the results. By collecting and reporting the home sites of patients, we hope to offer transparency around this potential source of bias.

The reporting of this study conforms to the STROBE guidelines for cross-sectional studies.^21^ This research was approved by the Research Ethics Board at Windsor Regional Hospital #20-370, Category A approval.

## Statistical Analysis

We summarized our finding by using common descriptive statistics. For categorical variables, we reported counts and their percentages, N (%), and displayed them by using histograms and pie charts wherever appropriate. On the other hand, interval variables (eg, age and turn-around times) were reported by using averages, medians and ranges (min to max). All patients were included consecutively. Patients were analyzed by disease site, site of home cancer site and referral to, and accrual to interventional clinical trials. There was no formal satisfaction survey of stakeholders. All comments for the program were unsolicited.

## Results

Before the COVID-19 pandemic forced almost all clinical trials to suspend activity in Canada, 118 Canadian patients used the CTN service between March 3, 2019, and March 12, 2020. Here we report the early findings of this program.

### Demographics

**Figure 2.**
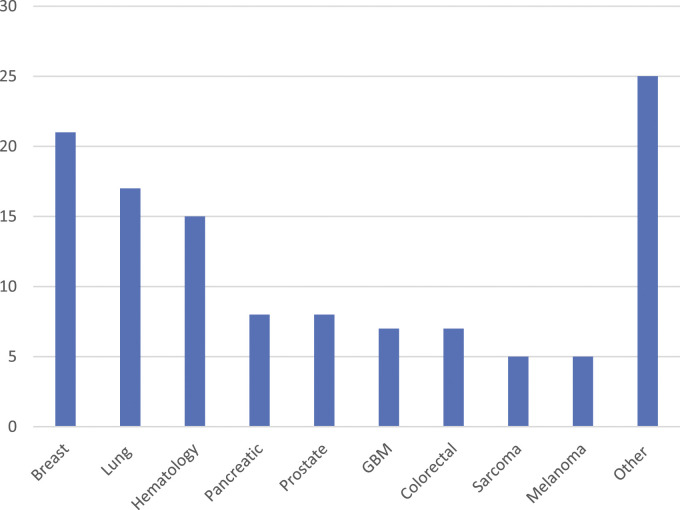
Number of patients referred to CTN by disease site.

Median age of patients using the CTN service was 46.5 (range of 29 – 81) years. Forty-nine percent of referred patients were female and 51% were male. Median number of prior lines of therapy was 2 (range of 0 – 9). Ninety-four percent of patients referred to the CTN were stage IV or relapsed refractory. The most common cancers occurring in greater than 10% of patients were hematologic malignancies: 13% (7 lymphoma, 4 multiple myeloma, 4 other)**;** cancers of the breast 18%; lung 14% (Figure 2). Twenty-six per cent of referred patients were from outside of the CTN pilot site, Windsor Regional Cancer Program. The majority of referrals were from small to medium sized cancer centres (86%) and all referrals were to clinical trials at larger centres. Seventy-four percent were from the host site.

At the onset of the CTN program, the average CTN turn-around time from physican or patient referral to generation of a summary report was 6-7 days. With experience, this time was reduced to approximately 1 day from referral to report. Physician and patient support received for this program has been positive, even though no formal evaluation of the program was performed. All comments were unsolicited. Patients have been favorable and very grateful for the help in searching for trials. Patients expressed gratitude for ‘all had been done’ in helping search for trials and the added assurance that and they were not missing opportunities in treating their disease. Physician engagement was positive at the host site, with thirteen of the fifteen oncologists providing a median of 7 referrals per physician per year (range of 1 – 21 referrals per physician). Thirteen other sites across Canada also used this service with increasing frequency being noted over time.

**Figure 3.**
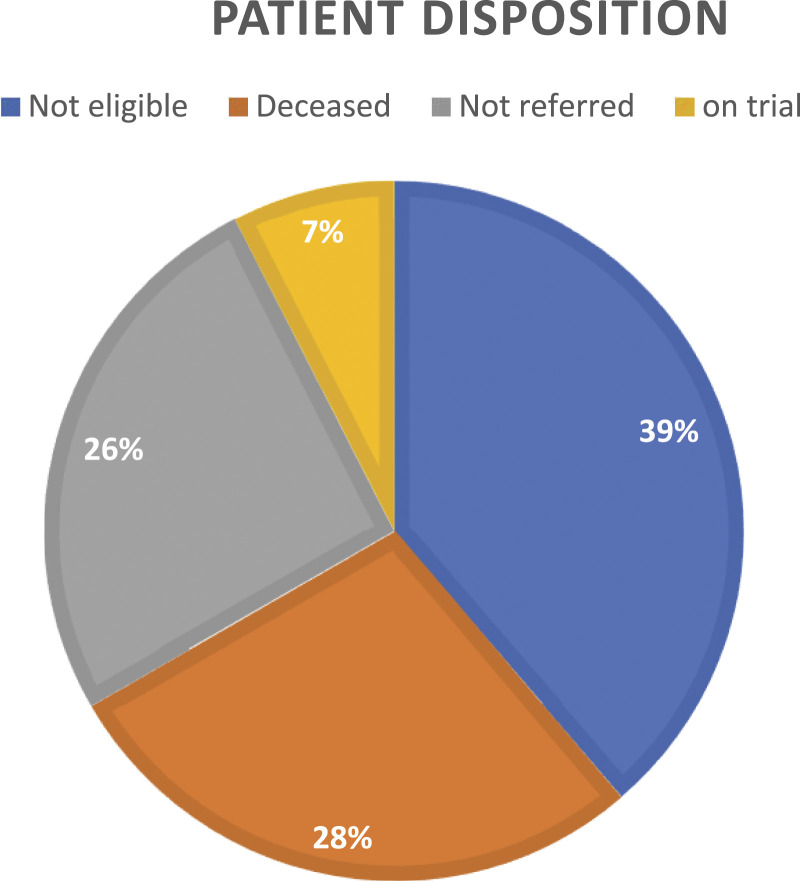
Disposition of patients with known follow-up referred to the CTN program.

In total, 93 (79%) referred patients have follow-up for clinical trial participation and 25 patients do not have follow-up and their data was excluded from further analysis. Patients from centres outside of the pilot site had no formal follow-up strategy. Twenty-nine of the 93 (31%) patients were referred for an interventional clinical trial with 7 (7.5%) ultimately enrolled All of the referrals that were identified by the CTN program were from the pilot site to larger centres. From the pilot site, all 29 patients were referred to identified clinical trials at larger sites in Ontario or Michigan. The absence of an available trial was the biggest challenge in 38.7% of patient cases. In 24 cases (26%) potential trials were identified but no referral was made. This was for various reasons, including patient choice, physician choice, and alternate therapy being identified. Moreover, as of February 2020, 27.9% of referred patients had died before they were referred or accrued to a clinical trial. In this group, the median time from referral to the CTN to death was 109 days (range of 3 – 188 days) (Figure 3).

The largest group of patient cases were those deemed not eligible for any matched trials. This number excludes those who declined before they could be referred for a clinical trial. Eight per cent of patients were not currently looking for clinical trials at the time of referral but rather investigating future options. Five per cent of patients were enrolled on trials outside of their own centre. In 2% of patients, the patient was referred to a trial listed on clinicaltrials.gov, but the trial was no longer open. Two patients found their own trial independent of the CTN program and were referred outside of this program.

## Discussion

In the first year, the CTN program has been successful in referring one third of patients who used the program to available trial opportunities and facilitating 7% of these patient accruals onto interventional clinical trials. All of the successful enrollments onto interventional trials were from smaller centres to larger centres.

Patients with rarer cancers such as Glioblastoma Multiforme (GBM), sarcomas and pancreatic cancers were more highly represented than those with more common cancers. As well, it has been previously demonstrated that patients with central nervous system (CNS) tumors are more motivated to enter trials.^22^ Participation by disease site in the CTN was similar to the clinical trials matching program sponsored by the American Cancer Society.^19^ This may be explained by the fact that smaller centres would be less likely to open trials for smaller disease sites. (Table 1) Although these are considered small patient groups, the numbers were as large as patients with colorectal cancer, prostate cancer and half as large as breast cancer patients looking for trials. This finding could influence smaller, community cancer centres in their trial decisions for this patient group, as we recognize that these smaller disease site groups as highly motivated proponents for clinical trials.

**Table 1.** Comparison of incidence of cancer type in Canada with the per cent of those referred to the CTN program by disease site.

Cancer Type	% Incidence of all Cancers in Canada	% Of Those Referred to the CTN
Breast	25.0	18
Lung	13.0	14
Colorectal	12	6
Prostate	10.3	7
Hematological	10.0	13
Melanoma	3.5	6
Pancreatic	2.7	6
GBM/CNS	1.3	6
Sarcoma	0.7	6

Almost one quarter of patients were referred within 3 and a half months of their death. This same finding was documented by the American Cancer Society program and additionally found that the most common reason for not participating was low energy and poor performance status.^19^ Gathering more information on the patient disease course will help understand this phenomenon.

Almost 40% of patients were not eligible for any clinical trial. While this result is similar to other reports in the literature where a noted lack of available trials was between 33 – 60%, it was nonetheless disappointing given CTN searches did include trial portfolios at the larger academic centres.^8,12,23,24^ A multi-pronged approach will be required to address this problem that includes the CTN as well as other unique initiatives for implementing decentralized trial delivery such as digital platforms that enable virtual trial activities, as well as the 3CTN-led Canadian Remote Access for Clinical Trials (CRAFT) developed to enable patients’ community healthcare teams to support their involvement in clinical trials.^25^

Although reported satisfaction was high, 25% of patients or physicians did not follow through with the list of potentially available clinical trials. Improving this metric will require additional investigation. Patient satisfaction was also high with unsolicited comments such as, “I felt that everything was being done.” Even though patients did not enter a clinical trial, they felt that they had done due diligence in exploring treatment opportunities and this offered a sense of peace to those patients and their families.

The CTN program showed promise in increasing patient accrual to interventional clinical trials, servicing primarily patients from smaller centres.^12^ Despite these strengths, the CTN approach has limitations. This was an observational study with the attendant limitations, and the numbers were relatively small. As well, we did not have follow-up data on 21% of patients. As well, while both turnaround time and quality of reporting improved during the pilot phase, the manual processes required present barriers to making further improvements in operating efficiency and restrict capacity for program growth and scaling. As this was a pilot program, and limited in manpower, limited resources were invested in advertisement, so many of the referrals were word-of-mouth, and therefore heavily weighted toward the pilot site, which was a medium sized cancer centre. Expansion of this program is ongoing with human resources expansion and expanded marketing of the program.

We are working with our information technology partners to streamline our data collection to address this barrier. We are also developing the standardization of the training and onboarding of the navigators to expand our reach. We will also formalize our follow-up strategies and developing formal assessments of the program from all stakeholders. In addition, we will be collecting patient equity, diversity and inclusion information to further assess our ability to improve access to clinical trials for all patients.

We feel that this program adds to the growing opportunities for those patients from smaller cancer centres who are under-represented in clinical trials.

## Conclusion

Lack of infrastructure to help patients navigate between smaller, community hospitals and larger academic hospitals in finding clinical trials is a significant barrier in optimal cancer care in Canada.

Our pilot evaluation of a personalized CTN program demonstrated high patient and physician satisfaction, and suggested an improvement in patient accrual to clinical trials. Capacity for program scale up and expanded human resources are being explored. Expansion of the CTN Program may help benefit patients across Canada through improved access and enhanced recruitment to interventional clinical trials.
